# Performance of Serum Quad Test in Screening for Fetal Down Syndrome in a Large-Scale Unselected Population in a Developing Country

**DOI:** 10.3389/ijph.2023.1605441

**Published:** 2023-04-05

**Authors:** Nitchanut Chaipongpun, Chanane Wanapirak, Supatra Sirichotiyakul, Fuanglada Tongprasert, Kasemsri Srisupundit, Suchaya Luewan, Kuntharee Traisrisilp, Phudit Jatavan, Sirinart Sirilert, Theera Tongsong

**Affiliations:** Department of Obstetrics and Gynecology, Faculty of Medicine, Chiang Mai University, Chiang Mai, Thailand

**Keywords:** developing country, down syndrome, prenatal diagnosis, prenatal screening, quadruple test

## Abstract

**Objective:** To assess the effectiveness of Quad test in the detection of Down syndrome (DS) in routine practice among a large-scale population and to compare the effectiveness of Quad test based on the Western reference model (WM) and that based on Thai reference model (TM).

**Methods:** Quad test was performed on 42,769 pregnancies at 14–21 weeks. The fetal risk of DS derived from Quad test was automatically computed based on WM and used in evaluating the effectiveness. Also, the fetal risk was calculated based on the TM.

**Results:** Of 39,740 women with complete follow-ups including 74 fetuses with DS, with WM, the detection and false positive rates were 81.1% and 7.2%, respectively, whereas the detection and false positive rates with TM were 87.8%, and 6.8%, respectively. According to ROC curves, the performance of Quad test based on TM was slightly but significantly better than that based on WM (AUC of 0.959 vs. 0.940, *p* = 0.001).

**Conclusion:** Quad test is highly effective in service settings and suitable for developing countries and the effectiveness is even higher when based on ethnicity-specific reference model.

## Introduction

Down syndrome (DS) is the most common aneuploidy with a prevalence of 1:319 to 1:1,000 live births (1); it is associated with poor quality of life and is one of the major public health problems (2), especially in developing countries. Several attempts have been made to prevent new cases of DS, and many screening tests are proposed (3). To date, among prenatal screening tests, NIPT (non-invasive prenatal testing) or cell-free fetal DNA analysis has the highest diagnostic performance, giving a sensitivity of more than 99% and false positive rate of less than 1% (3, 4). However, screening with NIPT is expensive, preventing it from being widely used in developing countries, where resources are very limited. According to our previous study on triple screen, the results of serum screening test seems to be promising (5). Accordingly, in 2015, the Thai government launched the national policy of fetal DS screening under the National Health Security Office (NHSO). All pregnant Thai women are to undergo Quad test screening free of charge. From our point of view, Quad test is one of the methods of choice for fetal DS screening in developing countries, where resources are limited. This is due to its advantages of simplicity, relative low cost, accessibility, feasibility of wide use, and non-requirement of expertise. In our practice, Quad test screening is routinely offered to pregnant women at 14–21 weeks of gestation at no cost. This strategy as well as a hospital network were developed, supported by the Thai government (NHSO). However, the true effectiveness of the screening has never been thoroughly evaluated, especially under service settings in developing countries. Though the test has been proven to be effective in research setting, mostly performed in developed countries, its true effectiveness or reproducibility is yet to be explored. Actually, several reports on the effectiveness of quad test in developing countries have been published, but most involved a small sample size, including a small number of fetuses with DS (6, 7). Additionally, as already known, ethnicity has a great impact on serum biomarker levels and accuracy of tests, and it cannot completely be corrected by ethnic factors, either in the first trimester or second trimester (8–11). Accordingly, our research team took advantage of this routine practice to evaluate the effectiveness of the strategy under the service settings of a large-scale unselected population and, also, to compare the diagnostic performance of Quad test based on Western reference models (WM) with ethnic correction and that based on Thai-specific reference models (TM). To the best of our knowledge, this is the largest prospective study on evaluation of the effectiveness of fetal Down syndrome screening in developing countries under service setting.

## Methods

A study of prospective screening test was undertaken at Maharaj Nakorn Chiang Mai Hospital (a tertiary care center and medical teaching school) and 64 network hospitals in the northern part of Thailand between 2016 and 2021. The study was conducted with ethical approval by the institutional review board, Chiang Mai University. The participants were enrolled with written informed consent after proper pretest counseling by the research team. In practice, the pregnant women attending antenatal care in the network hospitals underwent fetal DS screening using Quad test free of charge and the cases of high risk were referred to our hospital (Maharaj Nakorn Chiang Mai Hospital) for invasive prenatal diagnosis. The referral system together with the development of counseling teams, ultrasound standardization for gestational age, maternal blood sampling/collection, prenatal diagnosis and logistic system were supported by the NHSO. The study population was pregnant women attending antenatal care at our hospital and network hospitals at 14–21 weeks of gestation and underwent Quad test between January 2016 and December 2021. The inclusion criteria are as follows: 1) Thai ethnicity, 2) singleton pregnancy, and 3) gestational age of 14–21 weeks. The exclusion criteria are as follows: 1) fetuses with chromosomal abnormality other than Down syndrome or severe fetal anomalies requiring pregnancy termination, 2) unknown fetal status of Down syndrome, loss to follow-up, or miscarriage during the study, and 3) multi-fetal pregnancy.

### Quad Test Process

Pretest genetic counseling was provided to all of the participants by the project counseling team. The participants were interviewed regarding baseline characteristics prior to maternal blood sampling for Quad test, and their responses were stored in record forms. The collected data, which were also entered into the built-in software of the biomarker machine, included maternal age, gestational age (in weeks and days) at the time of blood sampling, ethnicity, residency, body weight, body mass index (BMI), history of smoking, pregestational diabetes mellitus, etc. The obtained blood specimens were handled as per standard protocol; they were immediately transferred to our lab and serum separation was done by centrifugation. The serum concentrations of the four biomarkers (alpha-fetoprotein: AFP, beta-human chorionic gonadotropin: b-hCG, unconjugated estriol: uE3, and inhibin-A) were measured by the same fully-automated bioassay machine (DELFIA^®^ Xpress system; Perkin Elmer, Waltham, MA, United States) with standard immunoassay screening kits of the four serum chemical markers. The immunoassays were run in batch to get rid of inter-assay variability.

### Fetal Risk Based on Western Reference Models

Automatically, the crude serum concentrations of the four biomarkers of all individuals were transformed to multiples of the median (MoMs) based on the Western reference models embedded in the built-in software, corrected by maternal body weight and ethnicity, smoking habit and diabetes status. The gestational age used in the calculation was primarily based on fetal sonographic measurement of crown-rump length or biparietal diameter in the first half of gestation. The final combined risk of fetal DS was automatically calculated by the LifeCycle software (PerkinElmer Risk Calculation engine), using maternal age as a baseline risk and multiplying it by the likelihood ratios of each serum biomarker, which is directly based on the relevant corrected MoM. A final risk of 1:250 or greater was considered as a high risk of fetal DS, indicating fetal chromosome work-up. In actual practice, the final risk reported by the LifeCycle software based on the Western reference models with ethnicity correction was used for counseling couples and as guidance for invasive prenatal diagnosis. The turnaround time for the Quad test results was usually within 2 weeks.

### Follow-Up for the Final Outcomes

The participants were followed-up for pregnancy outcomes, including fetal DS status and maternal complications, such as abortion, preterm birth, fetal growth restriction, preeclampsia, etc. Newborns of the participants were initially evaluated for DS based on clinical appearance by the neonatologists/pediatricians. Final diagnosis of fetal DS was confirmed by cytogenetic studies *via* amniotic fluid (amniocentesis), fetal blood analysis (cordocentesis) or postnatal chromosome study. The outcome of non-DS relied on cytogenetic studies in cases of invasive prenatal diagnosis or the conclusion by the neonatologists/pediatricians in cases of no clinical suspicion of chromosome abnormalities. Cytogenetic study in newborns was carried out only in cases clinically suspected of chromosomal abnormalities after assessment by the neonatologists/pediatricians.

### Statistical Analysis

#### Risk Based on Thai Reference Models

This risk was not used in actual practice but later calculated for research purpose. In fetal risk estimation, the MoMs of concentrations of the four biomarkers (AFP, b-hCG, uE3 and inhibin-A) were first computed as follows: the expected ln (biomarker concentration) for each individual based on Thai reference range model (12) was first determined, using gestational age in days and maternal body weight (Kg) as independent parameters, followed by converting the expected ln (biomarker concentration) to the expected biomarker concentration of each individual. The MoMs were then obtained by dividing the actual measured biomarker concentrations of each woman by the expected concentration. In the next step, log-Gaussian distribution of fetuses with DS and unaffected fetuses were developed, based on the MoM values described above. A likelihood ratio of each biomarker was then computed by dividing the density in fetal DS distribution by the density in the unaffected distribution. The final risk was determined by multiplying the risk by maternal age (priori risk) and by the likelihood ratios (LR) of the four biomarkers (*a priori risk x LR by AFP x LR by b-hCG x LR by uE3 x LR by inhibin-A*). The sensitivity or the detection rate of Quad test in predicting fetal DS was based on incidental detection using the risk of DS. Similar to the risk categorization used in the Western model, a risk of fetal DS of 1:250 or greater was defined as high risk, while a risk of lower than 1:250 was considered as low risk or not predicting fetal DS. The diagnostic performance (detection and false positive rates) was assessed for both models. Also, the effectiveness of the ROC curves of the two models in predicting fetal DS were compared, using Z-test. Sample size estimation was carried out based on the performance summarized by the American College of Obstetricians and Gynecologists (ACOG) (3), which reported that Quad test had a sensitivity of approximately 81% at a false positive rate of 5% for fetal DS screening among unselected pregnant women. Given a power of test of 80% at a confidence interval of 95% with allowable error in diagnosis of 0.1, this study needed at least 61 fetuses affected by DS. The prevalence of fetal DS is approximately 1:500 at a gestational age of 16 weeks. Therefore, the study needed a sample size of at least 30,732 tests. The statistical analyses were performed using Stata version 16 (StataCorp. 2019. Stata Statistical Software: Release 16. College Station, TX, United States: StataCorp LLC.) and the statistical package for the social sciences (SPSS) software version 26.0 (IBM Corp. Released 2019. IBM SPSS Statistics for Windows, Version 26.0 IBM Corp: Armonk, NY, United States).

## Results

**FIGURE 1:**
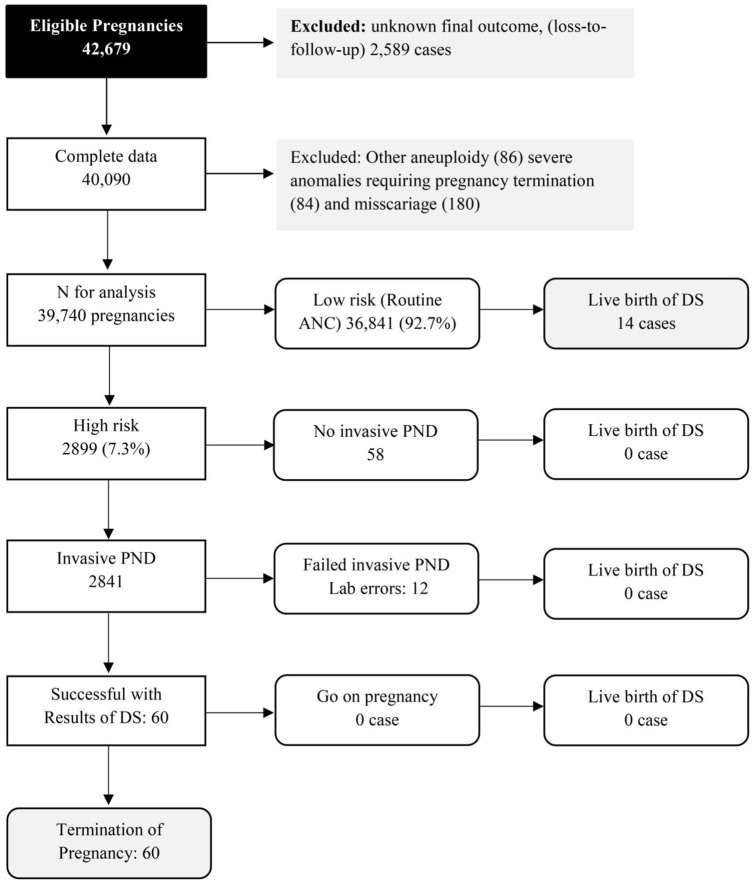
Flow chart of screening cascades in actual practice (Quad test based on western reference model with ethnic factor correction) (Down screening, Chiang Mai, Thailand, 2016–2021).

Of the 42,679 screened pregnancies, 40,090 pregnancies had known final outcomes of fetal DS status. Among them, 350 were excluded due to various reasons, such as fetal anomalies, miscarriage, as presented in Figure 1. The remaining 39,740 were available for analysis, including 74 pregnancies with fetal DS. The baseline characteristics of the participants are presented in Table 1. All of them were of Thai ethnicity, and the mean maternal age (±SD) at the time of blood sampling was 28.6 ± 5.7 years, including 12% of women with advanced maternal age (35 years or more). Most of the women (56.3%) were nulliparous. The mean gestational age at the time of blood sampling was 16.3 + 2.1 weeks of pregnancy. Only 0.2% of the women had smoking habit. According to Figure 1, 2.0% of pregnancies at high risk (58 out of 2,889 cases) did not undergo invasive prenatal diagnosis but all had unaffected fetuses. Note that among 74 pregnancies with fetal DS, 60 cases were confined in the high risk group and all of them chose to have pregnancy termination.

**TABLE 1:** Demographic data of the study population (Down screening, Chiang Mai, Thailand, 2016–2021).

Characteristics	Total (*n* = 39,740)	Down syndrome pregnancy (*n* = 74)	Normal pregnancy (*n* = 39,666)	*p*-value
Age (years)	28.66 ± 5.67	32.23 ± 6.57	28.66 ± 5.65	<0.001*
GA at sample collection (weeks)	15.8 ± 1.4	15.7 ± 1.3	15.8 ± 1.3	0.211
Maternal weight (kg)	58.6 ± 11.9	59.4 ± 11.9	58.6 ± 11.9	0.390
Nulliparous (%)	56.3%	59.3%	56.3%	0.332
GA at delivery (weeks)	38.3 ± 1.7	23.7 ± 7.5	38.3 ± 1.6	<0.001
Birth weight (gm)	2,888 ± 619	2,892 ± 611	582 ± 1,089	<0.001

Value presented as mean ± SD. *p*-value corresponds to independent *t*-test.

**FIGURE 2:**
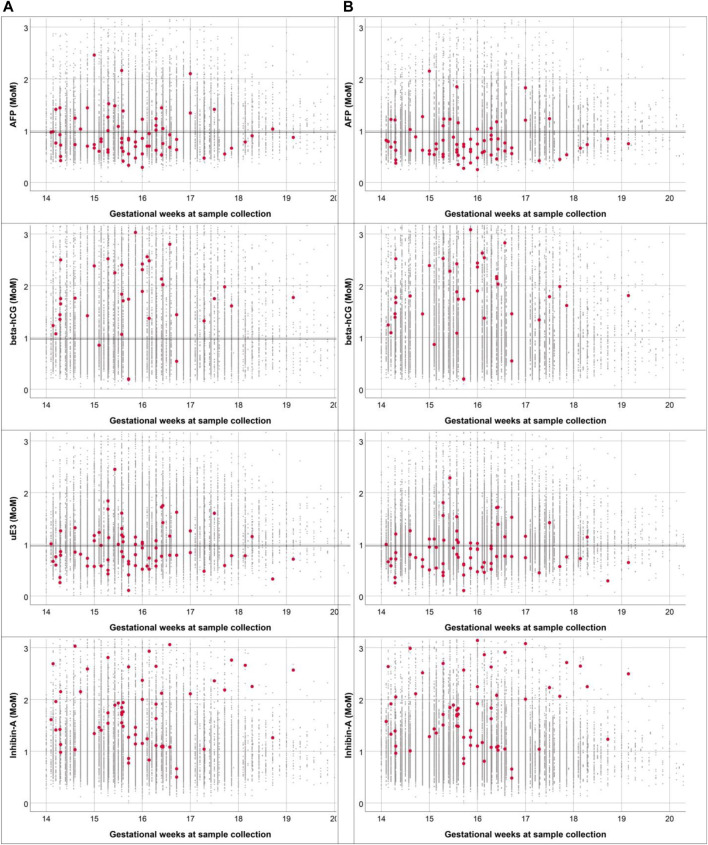
Comparison of distribution of multiple of medians for the four biomarkers based on Western reference model **(A)** and Thai reference model **(B)** (small black dot: normal fetuses; red dot: fetuses with Down syndrome) (Down screening, Chiang Mai, Thailand, 2016–2021).

**FIGURE 3:**
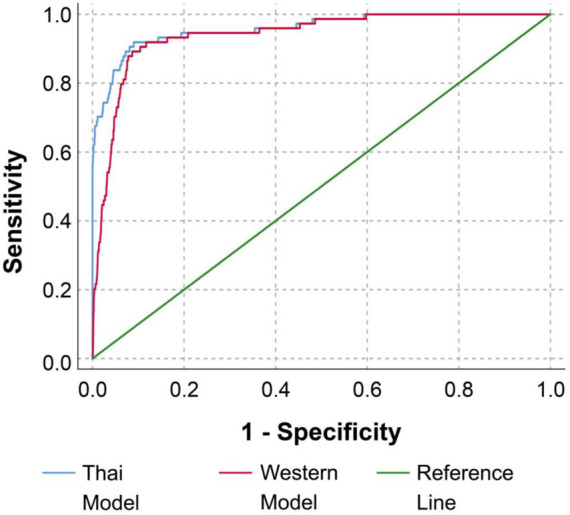
Receiver operating characteristic curves for diagnostic performance of Quad test based on Western reference model with ethnic correction and Thai reference model (Area under curve 0.940 vs. 0.959 for Western reference model vs. Western reference model, respectively, *p* < 0.001) (Down screening, Chiang Mai, Thailand, 2016–2021).

The differences in the patterns of concentration distribution of all biomarkers between pregnancies with fetal DS and unaffected pregnancies were observed to be similar in both Quad tests (based on WM and TM), as shown in Figure 2. The medians of AFP and uE3 are significantly lower in pregnancies with fetal DS compared with those of unaffected fetuses, whereas the medians of hCG and inhibin A are significantly higher in pregnancies of fetuses with DS compared with unaffected pregnancies. It is worthy of note that the MoMs of the four biomarkers derived from TM are slightly but significantly different from those derived from WM with ethnic correction, as shown in Table 2. The medians MoMs of the four biomarkers derived from TM are closer to 1 MoM than those derived from WM with ethnic correction. The diagnostic indices of Quad test based on WM with ethnic correction and TM are presented in Table 3. With WM, the detection and false positive rates were 81.1% and 7.2%, respectively, whereas those of TM were 87.8% and 6.8%, respectively. According to the ROC curves, the diagnostic performance of Quad test based on TM was slightly but significantly better than that based on WM with ethnic correction (AUC of 0.959 vs. 0.940, *p* = 0.001), as presented in Figure 3.

**TABLE 2:** Median and IQR of serum analyses based on Western specific reference model (WM) with ethnic correction compared with Thai specific reference model (TM) in both Down syndrome pregnancy and normal pregnancy (Down screening, Chiang Mai, Thailand, 2016–2021).

	Model	Down syndrome pregnancy (*n* = 74)	Normal pregnancy (*n* = 39,666)	*p*-value*
AFP	WM	0.84 (0.65, 1.05)	0.97 (0.77, 1.22)	<0.001
	TM	0.71 (0.55, 0.87)	0.99 (0.80, 1.23)	<0.001
	*p*-value **	<0.001	0.001	
Beta-hCG	WM	2.92 (1.75, 4.42)	0.99 (0.67, 1.5)	<0.001
	TM	2.96 (1.78, 4.49)	1 (0.68, 1.52)	<0.001
	*p*-value**	0.028	0.001	
uE3	WM	0.85 (0.62, 1.15)	1.09 (0.87, 1.36)	<0.001
	TM	0.79 (0.6, 1.08)	1.02 (0.81, 1.28)	<0.001
	*p*-value**	<0.001	<0.001	
Inhibin-A	WM	1.80 (1.22, 2.65)	0.95 (0.73, 1.25)	<0.001
	TM	1.75 (1.15, 2.63)	0.96 (0.75–1.26)	<0.001
	*p*-value**	<0.001	0.044	

*p-value derived from Mann-Whitney test for comparison between fetuses with DS and unaffected fetuses; **p-value derived from Wilcoxon-Signed rank test for comparison between Quad test based on WM with ethnic correction and that based on TM.

**TABLE 3:** Diagnostic performance in using Quad test based on Western specific reference model with ethnic correction and Thai-specific reference model (Down screening, Chiang Mai, Thailand, 2016–2021).

	Down syndrome pregnancy (*n* = 74)	Normal pregnancy (*n* = 39,666)	Total (*n* = 39,740)	Sensitivity (95% CI)	Specificity (95% CI)	False positive rate (95% CI)
Western model with ethnic correction						
High risk	60 (81.1%)	2,839 (7.2%)	2,899 (7.3%)	81.1 (72.2–90.0)	92.8 (92.6–93.1)	7.2 (6.9–7.4)
Low risk	14 (18.9%)	36,827 (92.8%)	36,841 (92.7%)			
Thai-specific reference model						
High risk	65 (87.8%)	2,711 (6.8%)	2,776 (7%)	87.8 (80.4–95.3)	93.2 (92.9–93.4)	6.8 (6.6–7.0)
Low risk	9 (12.2%)	36,955 (93.2%)	36,964 (93%)			

## Discussion

The essential insights gained from this study are as follows: 1) If each serum biomarker concentration of an unaffected woman or the same actual concentration of each serum biomarker is transformed to MoMs with different reference models, each MoM value will be different. The value is closer to 1 MoM when Thai-specific model is used. The findings imply that Quad test based on TM is more suitable for Thai population than that based on WM with ethnic correction. 2) Quad test either based on WM with ethnic correction or TM provides acceptable detection rate (greater than 80%), though the false positive rate is slightly high (greater than 5%). In other words, Quad test is effective or very helpful in actual practice (service setting) or reproducible when applied to a large-scale unselected population in developing countries. 3) Quad test based on TM gives a slightly but significantly higher area under curve. Similarly, the detection rate is higher, and the false positive rate is lower compared with Quad test based on WM. The findings imply that using ethnicity-specific model improves the performance of Quad test.

### Application

We provide solid evidence that the national policy of free Quad test, either based on built-in WM with ethnic correction or ethnicity-specific reference model, is highly effective; more than 80% of fetuses with DS will be prenatally diagnosed in developing countries, comparable with those reported in developed countries (3, 13). In contrast to most previous studies that were conducted under the circumstance of research, our results represent the effectiveness of the strategy under the circumstance of service setting. Moreover, whereas NIPT seems to be the most attractive and effective test (3, 14), it is impossible to implement it free of charge in developing countries for all pregnant women. Therefore, evidence from this study supports the assertion that Quad test is attractive and feasible for developing countries, though cost-effectiveness and cost-benefit are yet to be explored. Furthermore, Quad test is relatively simple in terms of management in large-scale use, in contrast to integrated tests and sequential serum biomarker screening (both stepwise and contingent screens), which are complicated and rather impractical. Finally, it is superior to the first trimester serum screening since many women have their visit for antenatal care after first trimester. In addition to its high effectiveness, Quad test has extra-advantages, including its simplicity, relatively low cost, high accessibility, feasibility of wide use, and non-requirement of expertise.

### Strengths

The strengths of this study are as follows: 1) The results are derived from a large-scale unselected population under service setting, representing the results in actual practice. 2) All newborns, both high risk and low risk, were evaluated for DS by the neonatologists/pediatricians in the research team. 3) All blood samples were tested to obtain measurements of the biomarkers in the same laboratories and same settings. 4) The participants were highly homogeneous in terms of ethnicity (Thai), leading to high reliability in representing the specific population. 5) The research was conducted with financial support by a non-profit organization with no conflict of interest. 6) The Thai reference models used in this study were constructed from a population group of the same ethnicity and geographical area.

### Weakness

The weaknesses of this study are given: 1) The transportation or logistic system can cause a problem of reliability of biomarker measurement. However, we were aware of the effects of transportation and temperature, which can affect the biomarker concentrations, as suggested by our preliminary study (15). Thus, we have always ensured adequate care in handling blood samples. 2) Other aneuploidies such as trisomy 13 and trisomy 18 were not included in the analysis. However, such aneuploidies are not considered as a major problem in developing countries because of their incompatibility with life. 3) The cost-effectiveness or cost-benefit of the strategy was not evaluated. 4) The large absolute number of cases with DS that could be reduced in a certain population as seen in this study may not be reproducible in populations which have no national policy of free test since many women might not take the test.

### Conclusion

Quad test, either based on WM with ethnic correction or ethnicity-specific model, is effective, helpful and reproducible in actual practice for screening fetal DS when applied to a large-scale unselected population in developing countries. Moreover, the performance of Quad test could be significantly improved when the test is based on ethnicity-specific reference models. This study provides solid evidence that Quad test based on TM is highly effective, giving a sensitivity of greater than 85% at a false positive rate of approximately 7% among Thai population. In developing countries, adopting free Quad test based on ethnicity-specific reference model as a national policy seems to be attractive and feasible. Nevertheless, the gap of knowledge which first needs to be fulfilled is that the cost-benefit and cost-effectiveness are yet to be evaluated. Hopefully, our results can be used as a good base for cost-effectiveness or cost-benefit analysis of the screening strategy in future.

### Highlights

• Quad test is highly effective in service settings and suitable for developing countries and the effectiveness is even higher when based on ethnicity-specific reference model.
• The national policy with free-of-charge Quad test in the study can encourage other populations in developing countries to develop their own policies.
